# Assessment of left ventricular function in horses with aortic regurgitation by 2D speckle tracking

**DOI:** 10.1186/s12917-020-02307-5

**Published:** 2020-03-20

**Authors:** A. Decloedt, S. Ven, D. De Clercq, F. Rademakers, G. van Loon

**Affiliations:** 1 Department of Large Animal Internal Medicine, Faculty of Veterinary Medicine, Ghent University, Ghent, Belgium; 2 Division of Cardiovascular Imaging and Dynamics, Department of Cardiovascular Sciences, KU Leuven, Leuven, Belgium

**Keywords:** Echocardiography, Valvular regurgitation, Strain, Strain rate

## Abstract

**Background:**

Aortic regurgitation (AR) may lead to left ventricular (LV) dilatation, cardiac arrhythmias and heart failure. Close follow-up of horses with AR is therefore paramount to detect onset of cardiac decompensation. The aim of this study was to examine whether two-dimensional speckle tracking (2DST) can be used to detect altered myocardial function in horses with chronic AR compared to control horses. Speckle tracking was performed on short axis and long axis images of the LV in 29 healthy Warmblood horses and 57 Warmblood horses with AR. Radial, circumferential and longitudinal strain, strain rate and displacement were measured for each segment separately and the average was calculated over all segments. Data generated from the apical segments were not included in the analysis.

**Results:**

Radial (SR) and circumferential (SC) strain were significantly higher in horses with moderate AR (average SR 75.5 ± 24.3%, SC 19.3 ± 3.2%) but not in horses with severe AR (SR 65.5 ± 26.2%, SC 16.3 ± 3.5%), compared to control horses (SR 54.5 ± 18.0%, SC 16.8 ± 3.0%). Longitudinal strain did not show significant differences, but longitudinal displacement (DL) was larger in horses with moderate (average DL 29.5 ± 4.1 cm) and severe AR (DL 32.4 ± 6.1 cm) compared to control horses (DL 25.7 ± 4.0 cm), especially in the interventricular septum. Diastolic longitudinal strain rate was lower in early diastole in horses with severe AR (0.93 ± 0.18/s) compared to controls (1.13 ± 0.13/s).

**Conclusions:**

2DST is able to detect altered myocardial motion in horses with AR, which showed significantly higher radial and circumferential strain. Further research is needed to determine whether these findings contribute to a more accurate diagnosis and prognosis in clinical cases.

## Background

Aortic regurgitation (AR) is quite common in horses, especially in older animals [1]. In most horses, the regurgitation is only mild, not associated with cardiac arrhythmias and remains stable for several years. However, some horses develop more severe regurgitation, left ventricular (LV) dilatation, ventricular arrhythmias and/or heart failure [2]. Follow up of these horses is paramount to detect early signs of decompensation [3]. Evaluation of myocardial function can be used to monitor the progression of the disease. Traditionally, fractional shortening (FS) or ejection fraction (EF) are used to estimate LV function, but in the compensated stage of the disease, FS and EF are unaltered [4, 5]. Tissue Doppler imaging (TDI) is an ultrasound technique that has been validated in horses [6] and is able to detect alterations in radial myocardial function in horses with different grades of AR [7]. However, since apical images cannot be obtained in horses, longitudinal myocardial function cannot be evaluated by TDI. In human medicine, longitudinal function has been shown to be the best marker of subtle myocardial dysfunction [8, 9].

2D speckle tracking (2DST) is an ultrasound technique that tracks speckles in the myocardial wall and allows evaluation of radial, circumferential and longitudinal wall deformation in horses. The technique has been shown to be feasible in horses, although diastolic measurements show a higher variability than systolic measurements [10–12]. 2DST has also been used as an indicator of regional or global LV function under various circumstances in horses [13–16]. In human medicine, 2DST has been used to detect LV systolic dysfunction in patients with AR [17]. However, as strain values are affected by loading, hyperdynamic deformation might be expected in the compensated stage of AR similar to the findings in dogs with mitral regurgitation [18].

The aim of this study was to investigate whether 2DST is able to detect changes in radial, circumferential or longitudinal LV function in horses with different stages of AR severity, compared to healthy control horses.

## Results

Descriptive statistics of all groups are listed in Table 1. There was a significant difference in age between control horses and horses with AR, with the controls significantly younger. Bodyweight, height at withers, heart rate, FS and EF were not significantly different between groups.

**Table 1:** Patient characteristics

	Control	Mild	Moderate	Severe	All AR
Number of horses	29	23	21	13	57
Age (years)	8 ± 4^a^	14 ± 6^b^ *P* = 0.001	15 ± 6^b^ *P <* 0.001	16 ± 6^b^ *P* < 0.001	15 ± 6
Body weight (kg)	566 ± 48	548 ± 61	542 ± 72	559 ± 78	548 ± 68
Height at withers (cm)	168 ± 4	166 ± 6	167 ± 7	169 ± 8	167 ± 7
Heart rate (beats/min)	40 ± 7	40 ± 6	39 ± 6	42 ± 10	40 ± 7
LVIDd (500) (cm)	11.1 (9.9–13.5)^a^	11.2 (9.5–13.2)^a^	12.4 (10.7–13.5)^b^ *P <* 0.022	13.7 (12.4–18.9)^b^*P* < 0.001	12.2 (9.5–18.9)
LVIDs (500) (cm)	6.4 (4.6–8.1)^a^	6.4 (4.4–8.1)^a^	7.3 (6.0–8.5)^b^*P <* 0.027	8.5 (6.1–10.8)^b^*P* < 0.001	7.1 (4.4–10.8)
FS (%)	43 ± 6	44 ± 7	41 ± 5	39 ± 11	41 ± 7
EF (%)	72 (49–83)	71 (50–86)	69 (61–76)	70 (39–80)	70 (39–86)

LVIDd (500), left ventricular internal diastolic diameter scaled for bodyweight; LVIDs (500), left ventricular internal systolic diameter scaled for bodyweight; FS, fractional shortening; EF, ejection fraction. Different superscripts indicate significant (*P* < 0.05) differences between groups (^a, b^). Variables with normal distribution are reported as mean ± standard deviation. Variables with non-normal distribution are reported as median (range)

Table 2 shows the results of systolic peak values and average values of the longitudinal, circumferential and radial measurements. SL did not show significant differences, but the group of horses with severe AR showed substantial individual variation, with some horses showing values lower than those found in the control group. In the IVS, longitudinal displacement was higher in horses with moderate to severe AR. A significantly higher average SR and SC was found in horses with moderate AR. SrR_S_ of the LVFW was higher in horses with moderate AR. SC of the cranial LVFW was significantly higher in horses with moderate AR.

**Table 2:** Segmental and averaged systolic peak values of 2DST measurements

		Control	Mild	Moderate	Severe
SL_G_	basSept	20.7 ± 3.7	20.5 ± 3.5	22.9 ± 4.3	22.6 ± 4.8
(%)	midSept	21.7 ± 2.6	21.2 ± 3.4	23.3 ± 3.0	22.9 ± 4.7
	midLat	19.7 ± 4.2	19.7 ± 4.5	20.9 ± 5.1	17.2 ± 5.7
	basLat	19.3 ± 4.1	20.4 ± 5.1	19.6 ± 6.0	17.7 ± 8.7
	average	20.3 ± 2.5	20.5 ± 2.7	21.7 ± 2.8	19.9 ± 5.2
SrL_S_	basSept	0.82 ± 0.14^a^	0.85 ± 0.17^a, b^	0.97 ± 0.24^b^ *P* = 0.036	0.92 ± 0.16^a, b^
(s^−1^)	midSept	0.86 ± 0.14	0.87 ± 0.14	0.94 ± 0.14	0.88 ± 0.11
	midLat	0.91 ± 0.15	0.86 ± 0.13	0.97 ± 0.17	0.89 ± 0.13
	basLat	1.05 ± 0.19	1.08 ± 0.16	1.07 ± 0.18	1.18 ± 0.37
	average	0.91 ± 0.12	0.91 ± 0.11	0.99 ± 0.12	0.96 ± 0.12
DL	basSept	30.7 ± 4.9^a^ *P* < 0.001	33.4 ± 6.0^a, b^ *P* = 0.001	36.9 ± 5.1^b, c^ *P =* 0.002	41.7 ± 8.2^c^
(mm)	midSept	17.6 ± 4.6^a^ *P* < 0.001	20.0 ± 3.7^a, b^ *P* < 0.001	22.9 ± 4.2^b, c^ *P* < 0.001	26.6 ± 4.7^c^
	midLat	20.6 ± 4.0^a, b^	19.5 ± 7.2^a^	22.4 ± 5.0^a, b^	24.7 ± 5.8^b^ *P* = 0.044
	basLat	33.9 ± 6.2	34.7 ± 9.3	35.7 ± 7.7	37.6 ± 9.9
	average	25.7 ± 4.0^a^ *P* < 0.001	26.9 ± 5.3^a, b^ *P* = 0.008	29.5 ± 4.1^b, c^ *P* = 0.039	32.4 ± 6.1^c^
SC	Ant	18.3 ± 4.6^a, b^	15.6 ± 5.0^a^	20.4 ± 5.4^b^ *P* = 0.009	18.6 ± 3.3^a, b^
(%)	AntSept	24.9 ± 6.1	22.7 ± 6.1	22.0 ± 5.4	19.6 ± 6.8
	Inf	9.8 (1.3–22.1)^a^	13.3 (3.8–25.2)^a, b^	14.4 (3.4–34.2)^b^ *P* = 0.048	14.7 (7.2–25.5)^a, b^
	Lat	16.7 ± 5.7	17.0 ± 4.3	20.3 ± 6.2	17.7 ± 4.6
	Post	12.1 ± 5.2^a^ *P* < 0.001	18.4 ± 5.8^b, c^ *P* = 0.005	21.2 ± 7.5^b^	14.3 ± 8.3^a, c^ *P* = 0.024
	Sept	18.1 ± 5.3	17.4 ± 5.5	17.9 ± 4.7	14.7 ± 6.9
	average	16.8 ± 3.0^a^ *P* = 0.040	17.4 ± 3.1^a, b^	19.3 ± 3.2^b^	16.3 ± 3.5^a^ *P* = 0.047
SrC_S_	Ant	1.02 ± 0.21	0.97 ± 0.16	1.09 ± 0.23	1.08 ± 0.23
(s^−1^)	AntSept	1.18 ± 0.30	1.12 ± 0.24	1.18 ± 0.28	1.00 ± 0.32
	Inf	0.77 (0.43–1.07)^a^	0.85 (0.48–1.55)^a, b^	0.87 (0.60–1.51)^a, b^	0.93 (0.79–1.07)^b^*P* = 0.017
	Lat	1.07 ± 0.18	0.99 ± 0.23	1.18 ± 0.28	1.10 ± 0.24
	Post	1.06 ± 0.20	1.15 ± 0.24	1.25 ± 0.27	1.16 ± 0.25
	Sept	0.92 ± 0.22	0.91 ± 0.21	0.94 ± 0.21	0.77 ± 0.23
	average	1.00 ± 0.14	1.00 ± 0.14	1.09 ± 0.16	0.99 ± 0.19
SR	Ant	53.3 ± 18.9^a^	56.5 ± 19.7^a, b^	73.8 ± 23.4^b^ *P* = 0.007	64.2 ± 26.4^a, b^
(%)	AntSept	55.5 ± 17.6^a^	58.9 ± 20.1^a, b^	73.4 ± 21.8^b^ *P* = 0.036	76.2 ± 32.8^b^ *P* = 0.038
	Inf	53.6 ± 20.4^a^	57.1 ± 21.4^a, b^	75.8 ± 28.4^b^ *P* = 0.008	62.9 ± 23.3^a, b^
	Lat	54.6 ± 21.1^a^ *P* = 0.018	56.0 ± 21.7^a^ *P* = 0.048	76.4 ± 29.2^b^	59.4 ± 29.9^a, b^
	Post	52.7 ± 19.4^a^ *P* = 0.004	56.8 ± 21.7^a^ *P* = 0.042	76.0 ± 26.2^b^	62.4 ± 27.0^a, b^
	Sept	57.3 ± 18.2^a^	58.1 ± 23.7^a, b^	77.4 ± 26.0^b^ *P* = 0.023	71.8 ± 27.6^a, b^
	average	54.5 ± 18.0^a^ *P* = 0.006	57.2 ± 20.1^a^ *P* = 0.037	75.5 ± 24.3^b^	65.5 ± 26.2^a, b^
SrR_S_	Ant	1.84 (1.17–2.98)^a^	1.99 (1.36–3.37)^a, b^	2.35 (1.01–3.46)^b^*P* = 0.007	1.94 (1.28–4.01)^a, b^
(s^−1^)	AntSept	1.85 ± 0.51	1.98 ± 0.51	2.26 ± 0.64	2.16 ± 0.83
	Inf	1.86 (1.04–3.23)^a^	2.02 (1.37–3.23)^a, b^	2.41 (1.58–4.95)^b^*P* = 0.011	2.09 (1.27–3.50)^a, b^
	Lat	2.22 (1.26–9.09)^a^	2.06 (1.36–3.47)^a, b^	2.50 (1.55–4.86)^b^*P* = 0.049	1.96 (0.96–4.25)^a, c^*P* = 0.041
	Post	2.05 (1.15–6.86)^a^	2.05 (1.40–3.29)^a, b^	2.38 (1.56–3.19)^b^*P* = 0.021	2.05 (1.08–4.06)^a, b^
	Sept	1.95 ± 0.51	2.00 ± 0.55	2.34 ± 0.57	2.21 ± 0.70
	average	1.96 (1.19–3.65)	2.02 (1.31–3.29)	2.37 (1.48–3.70)	2.06 (1.17–3.92)

*SL*_*G*_ Longitudinal strain, *SrL*_*S*_ Systolic longitudinal strain rate, *DL* Longitudinal displacement, *SC* Circumferential strain, *SrC*_*S*_ Systolic circumferential strain rate, *SR* Radial strain, *SrR*_*S*_ Systolic radial strain rate. Different superscripts indicate significant (*P* < 0.05) differences between groups (^a, b, c^). Variables with normal distribution are reported as mean ± standard deviation. Variables with non-normal distribution are reported as median (range)

In Table 3 results for diastolic strain rate measurements are listed. Previous studies have demonstrated a high variability of segmental diastolic measurements, therefore only averaged results are reported. SrL_E_ and SrC_E_ were significantly lower in horses with severe AR compared to the control group. SrR_E_ and SrR_A_ showed no significant differences.

**Table 3:** Averaged diastolic peak values of 2DST measurements

(s^**−1**^)		Control	Mild	Moderate	Severe
SrL_E_	average	1.13 ± 0.13^a^	1.02 ± 0.17^a, b^	1.04 ± 0.12^a, b^	0.93 ± 0.18^b^ *P* = 0.001
SrL_A_	average	0.67 ± 0.17	0.75 ± 0.14	0.78 ± 0.15	0.72 ± 0.13
SrC_E_	average	1.23 ± 0.17^a^ *P* = 0.007	1.22 ± 0.17^a^ *P* = 0.016	1.30 ± 0.13^a^ *P* < 0.001	1.04 ± 0.20^b^
SrC_A_	average	0.57 ± 0.17	0.60 ± 0.16	0.66 ± 0.16	0.58 ± 0.15
SrR_E_	average	1.78 (1.09–3.22)	1.83 (0.94–2.94)	2.00 (0.83–3.86)	1.71 (0.99–3.34)
SrR_A_	average	1.49 ± 0.68	1.52 ± 0.47	1.93 ± 0.56	1.85 ± 0.74

*SrL*_*E*_ Early diastolic longitudinal strain rate, *SrL*_*A*_ Late diastolic longitudinal strain rate, *SrC*_*E*_ Early diastolic circumferential strain rate, *SrC*_*A*_ Late diastolic circumferential strain rate, *SrR*_*E*_ Early diastolic radial strain rate, *SrR*_*A*_ Late diastolic radial strain rate. Different superscripts indicate significant (*P* < 0.05) differences between groups (^a, b^). Variables with normal distribution are reported as mean ± standard deviation. Variables with non-normal distribution are reported as median (range)

## Discussion

This study examines the ability of 2DST to detect altered myocardial function in horses with different stages of AR severity compared to control horses. In horses with moderate AR, radial and circumferential strain were significantly higher compared to healthy horses. In horses with severe AR, the radial and circumferential strain values did not differ from control horses, but the early diastolic longitudinal strain rate was significantly lower.

Speckle tracking is an ultrasound technique that allows quantification of the myocardial strain in two dimensions by tracking the speckles in the ultrasound image. The manually determined ROI is automatically divided in segments and the software calculates deformation of the wall for all segments separately and for the global ROI. The software algorithm is designed to analyse images obtained in human patients. For longitudinal function, a human apical image algorithm is being applied to a parasternal long axis image at nearly 90 degrees to the apical plane. For radial and circumferential function, the LV segment designations are assigned by the algorithm based on the human heart orientation which is different from the equine anatomy. The effect of these differences is unknown as the commercial software algorithms are undisclosed. In addition, segmental measurements could be influenced by alterations of LV geometry or loading. Therefore, the clinical value of segmental strain and strain rate values remains unknown. These indices of myocardial deformation might indicate both effects of ventricular loading, as well as changes in myocardial contractility.

Speckle tracking allows evaluation of the longitudinal function of the LV and the feasibility of the technique in horses was demonstrated [11]. In human patients with AR, systolic longitudinal strain and strain rate are considered an important indicator of subtle myocardial dysfunction [8, 17, 19]. Our results showed no significant differences in longitudinal systolic strain and strain rate between control horses and horses with AR. However, we previously demonstrated that horses with AR have a larger stroke volume [4], so an increased longitudinal strain would be expected in these horses. This might be counteracted by decreased longitudinal function due to AR, similar to what is found in human medicine. Both effects would then result in an unaltered longitudinal strain and measuring longitudinal myocardial function might therefore not be a good marker of subtle dysfunction in horses with AR. However, two horses showed SL peak values which were clearly below those of the control horses. For one of these horses, follow-up was available. This horse died of sudden cardiac death within 1 year after the initial exam.

Horses with moderate to severe AR did have a larger longitudinal displacement, however the difference was only significant in the IVS. This is probably a result of the increased ventricular length in horses with AR. Systolic radial strain increased significantly in all segments, especially in horses with moderate AR. This is in accordance with results from a study using tissue Doppler imaging [7] and might be a compensation for the decreased longitudinal function. In horses with severe AR, the lower strain values might be an indication of onset of decompensation. Systolic radial strain rate showed similar results, with the most pronounced effect in the LVFW. Circumferential strain was significantly higher in horses with AR, especially in the cranial LVFW. Again it was the moderate group that showed most significant results. Similar to radial function, this may also be a compensation for the reduced longitudinal function. The hyperdynamic LV deformation in the group with moderate AR might also be indicating compensated AR, with the left ventricle compensating for the volume overload as found in dogs with mitral regurgitation [18]. This is also reflected by the hyperdynamic motion of the interventricular septum on 2D or M-mode images and by increased myocardial velocities measured by tissue Doppler in horses with increased preload due to valvular regurgitation [20]. Horses with severe AR did not show increased strain values compared to the control group, which might indicate the onset of decompensation in at least some of these horses.

Previous studies have demonstrated a moderate to high variability for diastolic segmental measurements with 2DST [10, 11]. 2DST images have a low frame rate (40 fps) and this might lead to undersampling of the fast diastolic movement of the myocardial wall. Averaged values of all segments have a low to moderate variability, therefore only averaged values were reported. Diastolic longitudinal function was altered in horses with AR. In horses with mild or moderate AR, SrL_E_ was not significantly different from control horses. This probably results from the combined effects of age and AR. Horses with AR were significantly older than control horses and with increasing age the ventricle becomes stiffer and less compliant, resulting in a slower early filling (lower SrL_E_) [20–22]. AR itself also has an effect on ventricular filling. The AR jet causes a rapid rise in LV diastolic pressures, resulting in a higher SrL_E_ [23]. In horses with severe AR, a significantly lower SrL_E_ was found and this might be an indication of onset of decompensation. However, without an age matched control group no definite conclusions can be drawn. Averaged diastolic radial strain rates showed no significant differences, however measurement of SrR_E_ and SrR_A_ have been demonstrated to have a moderate variability and thus results should be interpreted with caution [10].

This study was intended to screen for variables that might be of interest for detecting myocardial dysfunction in horses with AR. Therefore a large number of variables was included, which may lead to false positive results. A post-hoc Bonferroni correction was applied to correct for multiple comparisons, but results should be interpreted with caution and more research is needed to determine the clinical usefulness of the variables that showed significant differences in this study. Ideally, a longitudinal follow-up study should be performed to evaluate which variables have prognostic value to predict further LV dilatation, ventricular arrhythmias and/or heart failure.

The main limitation of our study was the significant difference in age between the horses with AR and the control horses, which reflected the fact that horses usually develop AR at older age. This may have confounded our results, since several indices of myocardial function might be age dependent [21–23]. A recent study in 57 Warmblood horses aged 3 to 30 years demonstrated no effect of age on 2DST derived peak strain values [24]. Remarkably, the peak early diastolic strain rate increased with age in this study. This is in contrast with our results and other studies in horses and other species, which showed a decrease of the early diastolic relaxation velocity with age [21–23]. These changes with age have been attributed to increased stiffness of the LV, but could potentially also be caused by altered loading or heart rate [25].

Horses were divided in groups of different AR severity partially based on subjective criteria. Currently, there is no gold standard for quantification of AR in horses. The criteria used in this study are commonly used in clinical practice [4], but might not accurately reflect true AR severity. Other potential confounding factors in our study include the small number of horses in each AR severity group and the difficulty for blinding the observer. Only 13 horses were included in the group of horses with severe AR, which may have resulted in insufficient power to detect changes in longitudinal strain.

The last limitation is related to the 2DST technique. Adequate image quality is paramount for accurate tracking of the speckles. The software also performs extensive smoothing of the curves [12, 26]. Visual assessment of image quality and tracking quality should always be performed before approving the results. 2DST depends on the ultrasound machine, transducer and off-line analysis software and settings, so results might not be interchangeable with those acquired by a different ultrasound machine or software [26].

## Conclusions

2DST is able to detect altered myocardial movement in horses with AR. Radial and circumferential function show more significant differences than longitudinal function. Further research is needed to determine whether these findings contribute to a more accurate diagnosis and prognosis in clinical cases.

## Methods

### Subjects

This is an observational cross-sectional study. All horses involved were privately owned horses. Twenty nine healthy Warmblood horses (13 mares, 13 geldings, 3 stallions) aged 8 ± 4 years with a bodyweight (BW) of 566 ± 48 kg represented the control group. These horses were recruited prospectively. In order to be included, control horses had to be deemed healthy according to their owner and trained regularly. The AR group consisted of 57 Warmblood horses with AR (22 mares, 26 geldings, 9 stallions) aged 15 ± 6 years with a BW of 548 ± 68 kg, presented at the Faculty of Veterinary Medicine (Merelbeke, Belgium) for cardiac examination. All control horses and 35 AR horses were also included in previous studies [4, 7]. None of the horses were receiving medications.

Physical examination including thoracic auscultation was performed in all horses to exclude other cardiovascular and respiratory diseases. The study was approved by the ethical committee of the Faculty of Veterinary Medicine and Bioscience Engineering (approval number EC2012_57). Owner informed consent was obtained for all horses.

After the study, all horses were discharged from the hospital.

### Echocardiography

Echocardiography was performed in all horses without sedation, using a GE Vivid 7 Dimension ultrasound with 3S phased array transducer (GE Healthcare, Diegem, Belgium) at a frequency of 1.6/3.2 MHz with simultaneous recording of an electrocardiogram. Images were stored for off-line analysis.

Standard 2DE, M-mode and color flow Doppler images were recorded. Horses with AR were not included if they had more than mild regurgitation of another cardiac valve. Valve regurgitation was subjectively graded taking into account the jet duration, the number of images in which the jet was visible and the area of the receiving chamber covered by the jet [1]. For 2DST examination from a right parasternal view a slightly modified four chamber image was recorded so that the mitral annulus was visible throughout the cardiac cycle. Due to the size of the equine heart, the apex could not always be visualized throughout the cardiac cycle in the four-chamber view, despite adaptations of the image tilt and probe angulation [11]. Left ventricular short axis images at chordal level were also recorded from a right parasternal view. In all images sector width was reduced to 55° to achieve a frame rate of at least 40 frames per second.

### Off-line analysis

**Fig. 1:**
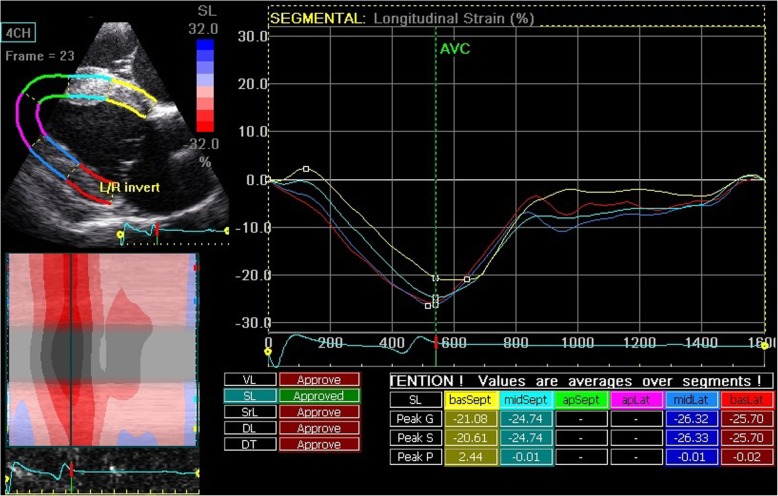
Right parasternal long axis four chamber image of the left ventricle. The image is optimized to keep the mitral annulus visible throughout the cardiac cycle. The region of interest is automatically divided into six segments (basSept, midSept, apSept for the interventricular septum; basLat, midLat, apLat for the left ventricular free wall). The segmental traces for longitudinal strain are displayed

**Fig. 2:**
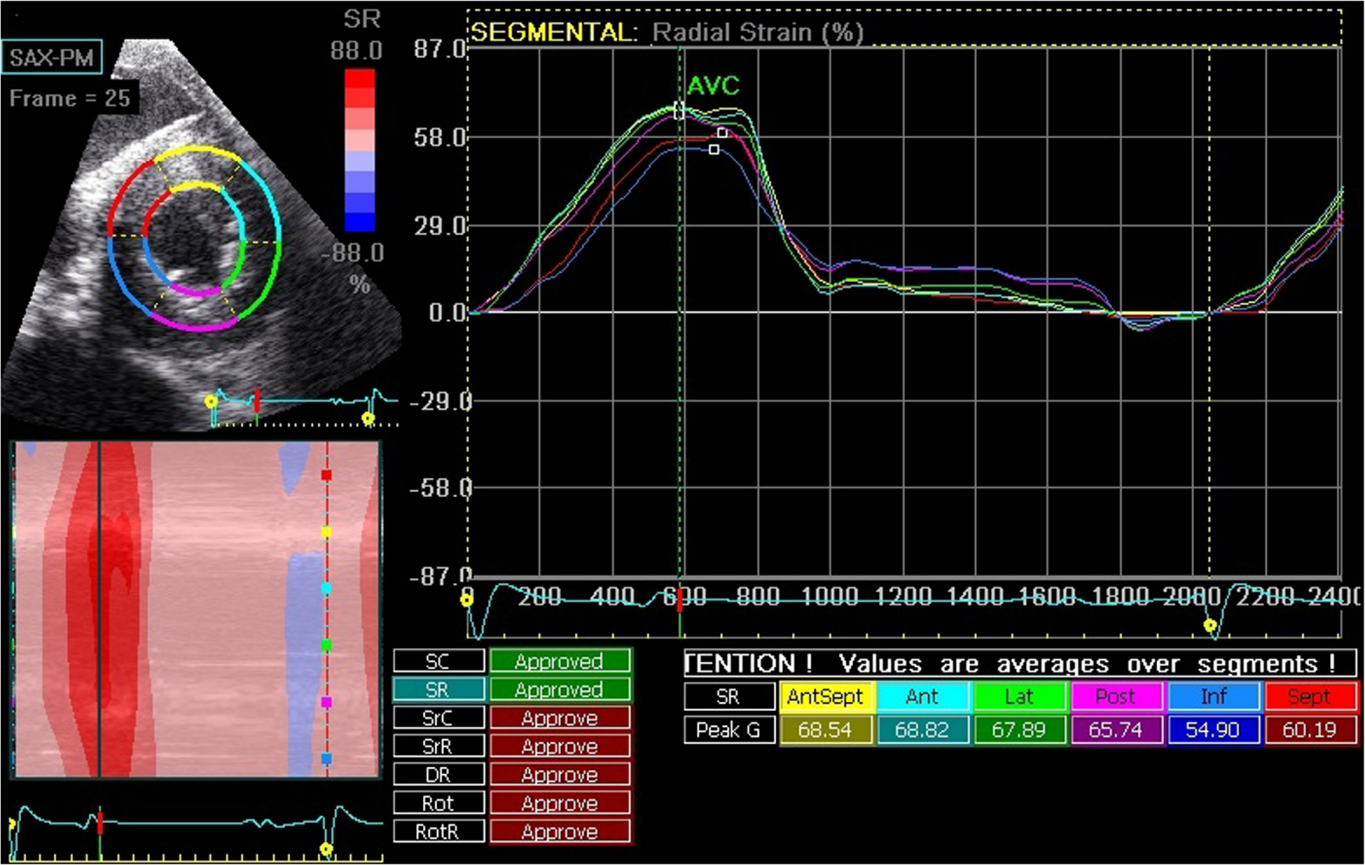
Right parasternal short axis image of the left ventricle, taken at end-systole. The region of interest is automatically divided into six segments (Sept and AntSept for the interventricular septum; Ant and Lat for the caudal left ventricular free wall; Inf and Post for the cranial left ventricular free wall). The segmental traces for radial strain are displayed

Off-line analysis was performed using dedicated software (EchoPAC Software Version 11.2, GE Healthcare, Diegem, Belgium). For each image, 3 cardiac cycles were analyzed and then averaged. End-diastole was defined as the peak R wave on the ECG. To evaluate the longitudinal LV wall deformation, the right parasternal four-chamber long axis images were analyzed and the region of interest (ROI) was set by tracing the LV endocardial border from the septal insertion of the mitral valve until the insertion point on the lateral myocardial wall. The width of the ROI was set to cover the myocardial wall but not the epicardium (Fig. 1) and was automatically divided by the software into 6 segments: basal (basSept), mid (midSept) and apical (apSept) part of the interventricular septum, and basal (basLat), mid (midLat) and apical (apLat) part of the LV free wall (LVFW). Data generated from the apical segments were not included in the segmental analysis but for the global measurements, those segments were included by the software. Therefore, global values are not reported in this study. To evaluate LV radial and circumferential wall deformation, short axis images (caudal right on screen) were analyzed. The ROI was set by tracing the endocardial border and adapting the width of the ROI. The software divided the ROI automatically in 6 segments based on the human heart orientation. In horses, AntSept and Sept represent the interventricular septum, Inf and Post represent the cranial LVFW and Lat and Ant define the caudal LVFW (Fig. 2).

Tracking quality of all images was calculated by the software and verified visually by the operator. When necessary, ROI was adapted manually until tracking quality was good [10, 11]. For the longitudinal motion, strain (SL), strain rate (SrL) and displacement (DL) curves were displayed. From the short axis images, curves for radial strain (SR) and strain rate (SrR) and for circumferential strain (SC) and strain rate (SrC) were derived. The timing of aortic valve closure (AVC) was automatically calculated by the software algorithm based on the segmental curves. Strain values were reported by the software as systolic peak values occurring before AVC and the absolute maximal peak value which could also occur after the calculated AVC. From the strain and displacement curves maximal segmental strain or displacement was measured and average strain over all segments was calculated manually. From the strain rate curves, segmental systolic (S), early diastolic (E) and late diastolic (A) peak values were measured and average strain rate over all segments was calculated [10, 11].

### Assessment of severity

All horses with AR were scored as having mild, moderate or severe AR. This scoring was done based on three criteria as described previously: LV dilatation, size of the regurgitant jet and the end-diastolic LV internal diameter (LVIDd) scaled to a BW of 500 kg [4]. The scoring method is explained in more detail in Additional file 1.

### Statistical analysis

Statistical analysis was done using commercially available software (SPSS Statistics version 25, Chicago, IL, USA). Distribution of all variables was verified by evaluation of the residuals by visual inspection, the Kolmogorov-Smirnov test and Shapiro-Wilk test. Levene’s test for equality of variances was performed to check homogeneity of variance across groups. Variables with normal distribution and homogeneous variances were evaluated by one-way ANOVA with Bonferroni correction to compare means of the four groups (control, mild, moderate and severe AR). A non-parametric Kruskal-Wallis test with post-hoc pairwise comparison using the Dunn test was applied to compare all non-normal distributed variables. Level of significance was 0.05.

Variables with normal distribution are reported as mean ± standard deviation (SD), non-normally distributed variables are reported as median (range).

## Supplementary information

**Additional file 1.** Scoring method for AR severity.

## Data Availability

The data supporting the conclusions of this article are included within the article (and its additional files). All datasets used and/or analysed during the current study are available from the corresponding author on reasonable request.

## Abbreviations

2DST: Two dimensional speckle tracking
A: Late diastole
AR: Aortic regurgitation
BW: Bodyweight
DL: Longitudinal displacement
E: Early diastole
EF: Ejection fraction
FS: Fractional shortening
IVS: Interventricular septum
LV: Left ventricle
LVFW: Left ventricular free wall
LVIDd: Left ventricular internal diameter at end-diastole
LVIDs: Left ventricular internal diameter at end-systole
ROI: Region of interest
S: Systole
SC: Circumferential strain
SD: Standard deviation
SL: Longitudinal strain
SR: Radial strain
SrC: Circumferential strain rate
SrL: Longitudinal strain rate
SrR: Radial strain rate
TDI: Tissue Doppler imaging
