# Supplementary material for: Assessment of left ventricular function in horses with aortic regurgitation by 2D speckle tracking
Source: BMC Vet Res. 2020 Mar 20;16:93. doi: 10.1186/s12917-020-02307-5 (PMC7085189; doi:10.1186/s12917-020-02307-5)
Supplement: Supplementary file 1 — Additional file 1. Scoring method for AR severity. [file 12917_2020_2307_MOESM1_ESM.docx]

**Supplementary information**: Aortic regurgitation (AR) severity scoring method

Scoring of AR severity was done based on 3 criteria [[1](#_ENREF_1)].

1. Left ventricular internal diameter (LVID), measured from a right parasternal short axis M-mode image at chordal level in diastole and corrected for bodyweight using the formulae of allometric scaling [[2](#_ENREF_2)]: LVID(500) = measured LVID/BW^1/3^ x 500^1/3^

| **LVID(500)** | **Points** |
| --- | --- |
| 12-13 cm | 1 |
| 13-14 cm | 2 |
| > 14 cm | 3 |

1. Subjective evaluation of the size and area of the regurgitant jet. The jet was visualized by Color Flow Doppler at 2.4 MHz and -5.0 dB gain. Velocity scale was set from +47 to -47 cm/s. The size of the jet was evaluated relative to the size of the left ventricular area.

| **Jet size** | **Points** |
| --- | --- |
| < 1/3 LV area | 1 |
| < 2/3 LV area | 2 |
| > 2/3 LV area | 3 |

1. Subjective evaluation of the left ventricular size from the four chamber view

| **Criteria evaluated** | **Points** |
| --- | --- |
| Normal ventricle, no signs of enlargement, normal shape of the apex. | 1 |
| Normally shaped ventricle, mild enlargement, normal shape of the apex. | 2 |
| Large ventricle with normal shape, apex slightly rounder than normal. | 3 |
| Large ventricle, apex with globoid aspect. | 4 |
| Severe enlargement with a very large rounded ventricle. | 5 |

Points scored for each criterium were added and classification was made based on this final score:

- More than 8 points: severe AR
- 5, 6 or 7 points: moderate AR
- Less than 4 points: mild AR

[1] Ven, S., Decloedt, A., Van Der Vekens, N., De Clercq, D. and van Loon, G. (2016) Assessing aortic regurgitation severity from 2D, M-mode and pulsed wave Doppler echocardiographic measurements in horses. *Vet. J.* **210**, 34-38.

[2] Huesler, I.M., Mitchell, K.J. and Schwarzwald, C.C. (2016) Echocardiographic Assessment of Left Atrial Size and Function in Warmblood Horses: Reference Intervals, Allometric Scaling, and Agreement of Different Echocardiographic Variables. *J. Vet. Intern. Med.* **30**, 1241-1252.
